# Microbiota composition and distribution along the female reproductive tract of women with endometriosis

**DOI:** 10.1186/s12941-020-00356-0

**Published:** 2020-04-16

**Authors:** Weixia Wei, Xiaowei Zhang, Huiru Tang, Liping Zeng, Ruifang Wu

**Affiliations:** 1 Department of Obstetrics and Gynecology, Peking University Shenzhen Hospital, Shenzhen, 518036 China; 2 Shenzhen Key Laboratory on Technology for Early Diagnosis of Major Gynecological Disease, Shenzhen, 518036 China; 3 BGI-Shenzhen, Shenzhen, 518083 China

**Keywords:** Endometriosis, 16S rRNA gene amplicon sequencing, Microbial community composition, Microbial distribution

## Abstract

Endometriosis (EMS) is a multifactorial disease that affects 10%–15% women of reproductive age and is associated with chronic pelvic pain and infertility. The pathogenesis of EMS has not been consistently explained until now. In this study, we involved 36 endometriosis patients and 14 control subjects who performed laparoscopic surgery due to gynecological benign tumor. The samples from lower third of vagina (CL), posterior vaginal fornix (CU), cervical mucus (CV), endometrium (ET) and peritoneal fluid (PF), were collected and sequenced by 16S rRNA amplicon. The continuous change of the microbiota distribution was identified along the reproductive tract. The flora in lower reproductive tract (CL, CU) were dominated by *Lactobacillus*. Significant difference of the community diversity began showing in the CV of EMS patients and gradually increased upward the reproductive tract. It indicates the microbiota in cervical samples is expected to be an indicator for the risk of EMS. This study also highlights the decreasing of *Lactobacillus* in vaginal flora and the increasing of signature Operational Taxonomic Units (OTUs) in transaction zone (CV) and upper reproductive tract (ET, PF) of EMS patients, which reflect the alteration of microbial community associated with EMS, participation of specific colonized bacteria in the EMS pathogenesis and relationship between microbiota and development of disease.

## Introduction

Endometriosis (EMS) is a condition in which cells similar to those in the endometrium, the layer of tissue that normally covers the inside of the uterus, grow outside of it [1]. It may cause severe primary dysmenorrhea, infertility and pelvic mass, which seriously affect the reproductive ability and life quality. According to previous study, EMS occurred in as high as 10%–15% women of reproductive age, and the incidence rate is increasing year by year [2–4]. Despite different hypotheses for the development of the EMS were reported, the pathogenesis of EMS is still hard to consistently explained even after a 300 years’ investigation [5–7]. Many studies have detected a variety of possible endotoxins in the peritoneal cavity of EMS patients that could regulate the pro-inflammatory reaction and promote the growth of endometriosis [7–9]. Lipopolysaccharide (LPS) is one of the endotoxins functioned via Toll-like receptor 4 (TLR4), which is produced by bacteria [10, 11]. Therefore, some scholars proposed “bacterial contamination hypothesis” as the key factor to the pathogenesis of EMS [12]. The previous study has been reported that the bacterial colonization is shown in the menstrual blood of women with EMS by the detection of *E. coli* [13]. Also, the bacterial endotoxins probably translocate from gut to pelvic cavity [14]. As a result, higher concentration of endotoxin (Lipopolysaccharide) are detected in the menstrual blood and peritoneal fluid [13]. In addition, through the culturing approach on endometrial samples, increased bacterial colonization is demonstrated in the intrauterine microbiota. Interestingly, the treatment of the gonadotropin releasing hormone agonist (GnRHa) shifts vaginal pH to higher than 4.5 by altering the bacterial community and increases the risk of endometriosis [15].

However, to further investigate and clarify the “bacterial contamination hypothesis”, the primary limitation is the low abundant of intrauterine microorganisms, and the exhaustive bacterial community composition is hard to address via traditional culturing method [15], not to mention most of the bacteria is uncultured according to the current technique [16]. Hence, based on the previous study, the understanding of the intrauterine microbial colonization still remains largely unexplored. In 2017, Chen et al. applied the new generation sequencing method of 16S rRNA gene amplicon to elaborate that the distinct microbial community was continuously harbored along the female reproductive tract, including cervical canal, uterus, fallopian tubes and peritoneal fluid [17]. Then, the microbial structure and function in the upper reproductive tract were revealed by metagenomic analysis [18]. These studies not only break the traditional concepts on uterine sterility, but also opens up new research methods for the study of female reproductive tract diseases, such as endometriosis.

In this study, we invited 16S rRNA amplicon sequencing method to study the flora distribution and bacterial community based on different taxonomic levels across the whole (upper and lower) reproductive tract of the EMS patients and non-EMS women. Through the comparative analysis, the alteration of the microbiota along the reproductive tract was evaluated, the EMS-specific bacterial species colonized was identified and the relationship between the flora and disease development was inferred. It provides a new way to explore the pathogenesis of endometriosis and offers a more comprehensive understanding of the bacterial factors on EMS.

## Materials and methods

### Study population

This study was approved by the Medical Ethics Committee of Peking University Shenzhen Hospital. Informed consent was completed for all subjects enrolled in the study. Fifty patients involved in this study were aged 23–44 years (31.47 years old in average) who took the laparoscopic surgery between December 2013 and July 2014 in Peking University Shenzhen Hospital due to benign gynecological diseases or pelvic endometriosis. Despite we included the same sampling cohort as our previous study, the current study focused on EMS patients and the association between microbiota and endometriosis phenotype. According to the pathology examination, they were divided into two groups: 36 cases for the study group, and 14 for control group. All of the study cases were pelvic endometriosis, and 16 cases were in I ~ II stage and 20 were in III ~ IV stage which determined according to EM staging method revised in 1985 by the American Fertility Association (Revised American Fertility Society, r-AFS). 14 subjects in the control group were performed laparoscopic surgery due to gynecological benign tumor, including 7 cases of ovarian teratoma, 4 cases of serous cystadenoma, 3 cases of uterine fibroids. Also, no endometriosis symptom was shown in control individuals confirmed both by surgery observation and pathology assay. All the women selected in this study have a regular menstrual cycle (28 ± 2.2 day/cycle) and followed exceptional rules including acute inflammation, malignant tumors and autoimmune diseases. In addition, they had no recorded of using hormonal drugs or antibiotics within 6 months and vaginal medications within 3 months.

### Sample collection and treatment

Samples were collected 3–7 days after the menstrual period (early follicular phase) from five different sites throughout the reproductive tract, 3 from lower sites and 2 from upper ones. Those located in lower reproductive tract included lower third of vagina (CL), posterior vaginal fornix (CU) and cervical mucus (CV), which collected before taking any therapeutic methods. A sterile swab rotated gently for 3 to 5 circles to obtain the secretions and stored in a 2 ml tubes (Chenyang Hua, Shenzhen). For the upper reproductive tract samples, endometrium (ET) and peritoneal fluid (PF) were taken during the operation. ET samples were obtained by injecting 2 ml of sterile saline to uterine cavity using a sampler (Huales Medical Machinery, Ningbo), then absorbing the uterine lavage fluid after 1 min and placing in the 5 ml tubes. During the surgery, about 10 ml peritoneal fluid from Douglas pouch were extracted as PF samples and placed in 15 ml centrifuge tubes. All samples were immediate pre-frozen in dry-ice after collection, and then transferred to - 80 °C for storage to avoid freezing and thawing.

In addition, negative control samples were collected carefully which use the same sampling tools and methods performed on saline instead. Rare biomass was detected from these samples and this information has been confirmed in our previous study [17].

### Sample microbial genome DNA extraction and detection

Total DNA of the sample was extracted using QiaGen QIAamp DNA Mini Kit (QIAGEN, Germany) and the quality was determined by a Qubit Fluorometer. The V4–V5 region of the 16S rRNA genes was amplified by PCR with universal primersV4-515F (5ʹ-GTGCCAGCMGCCGCGGTAA-3ʹ) and V5-907R (5ʹ- CCGTCAATTCMTTTRAGT-3ʹ). The purity and integrity of the PCR product were measured by 0.6% agarose gel electrophoresis. The DNA samples were stored at -20 °C for the further requirements.

## 16S rRNA amplification of V4-V5 region and Ion proton PGM platform sequencing

Amplification of the 16S rRNA V4-V5 region fragment were carried out using the total DNA by Ion torrent PGM sequencing platform for V5 → V4 reverse sequencing (BGI, Shenzhen).

### Data analysis

The raw data obtained by sequencing was analyzed by Mothur (V1.33.3) software, and the OTUs were subjected to species annotation.

## Results

### Microbial composition and distribution of the in the reproductive tract

**Fig. 1:**
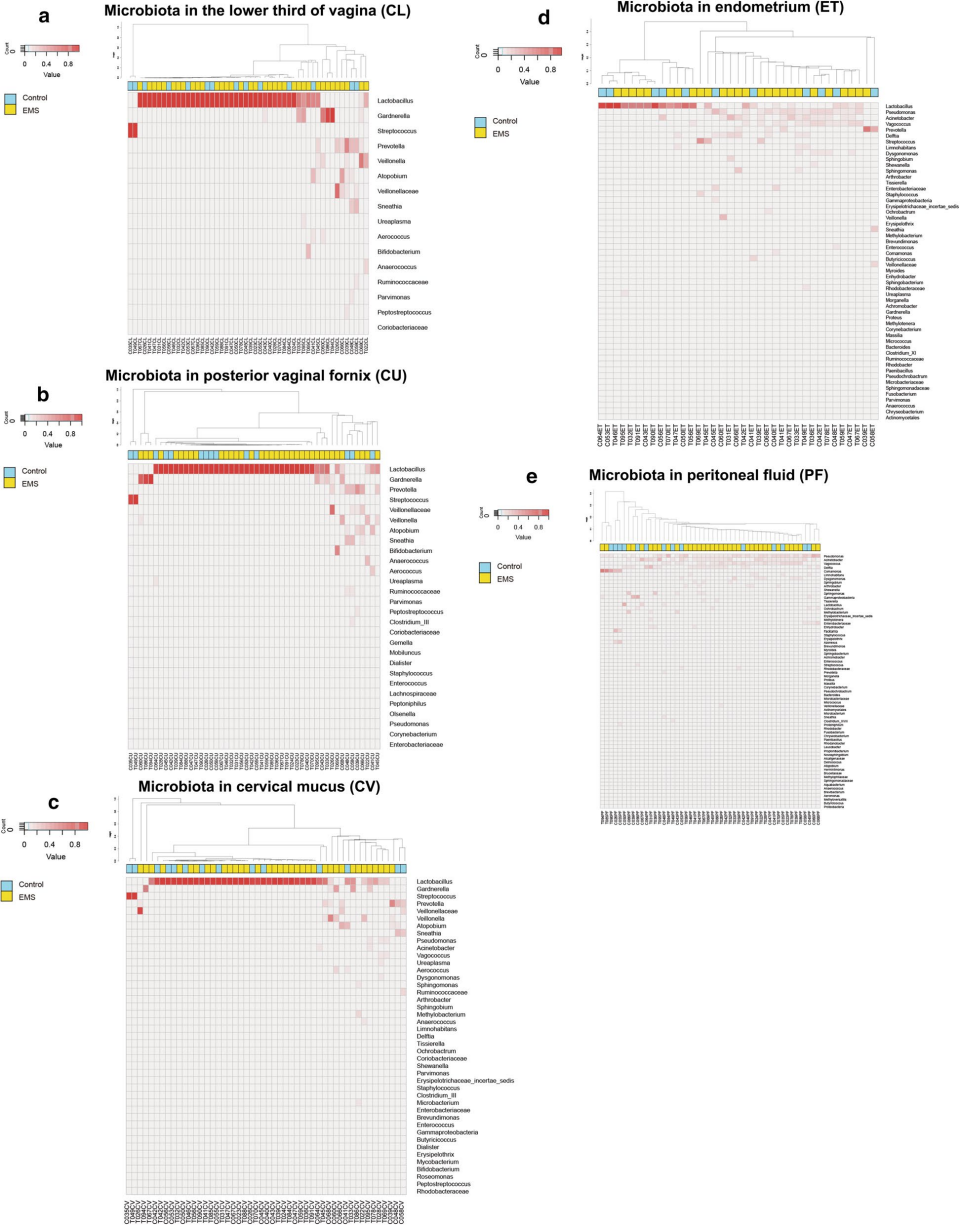
The distribution of the dominate bacterial species colonized in female reproductive tract were presented, including **a** vagina (CL), **b** posterior vaginal fornix (CU), **c** cervical mucus (CV), **d** endometrium (ET) and **e** peritoneal fluid (PF) of the EMS patient group and control group. The roman numerals labeled on the clades represent the classified flora types. The fade-in red cubes stand for the different abundance gradually from 0% to 100%

Samples on five different reproductive tract locations (CL, CU, CV, ET and PF) were systemically collected from 50 women in this study, which included 36 EMS patients and 14 women without any endometriosis symptom. We collected 50 samples for each location except ET ones only obtained from 26 EMS patients and 11 control individuals. As a result, 237 samples were subjected to the 16S rRNA gene amplicon sequencing. According to the dominant genus identified from the sample, we grouped them into the following seven types. The samples dominated by *Streptococcus* were determined as type I; *Lactobacillus* as type II; *Gardnerella* as type III; a mixture of *Prevotella*, *Veillonella*, *Atopobium*, *Veillonellaceae* and others as type IV; *Veillonellaceae* as type V; a mixture of *Pseudomonas*, *Acinetobacter*, *Vagococcus* and others as type VI and *Comamonas* as type VII. The microbiota in different site of the reproductive tract of every individual were presented in Fig. 1 with the abundance of the genera. In general, most of them in lower reproductive tract (CL, CU and CV) were dominated by *Lactobacillus* which belong to type II, while it totally changed in the upper reproductive tract. In ET and PT, higher diversity was presented, and type IV and V occurred more frequently.

The bacterial community distribution in CL was similar to CU samples, which consisted by type I, II, III and IV. For EMS cases, the numbers of types identified in CL were 0, 28, 3 and 5 respectively (Fig. 1a). While for CU, pretty similar distribution was showed as 0, 26, 3 and 7 (Fig. 1b). In the control cases, the distribution numbers in type I, II, III and IV were 2, 10, 0 and 2 for CL and 2, 9, 0 and 3 for CU respectively. The results indicated that the microbiota of the female lower reproductive tract was mainly dominated by *Lactobacillus* (type II) either in EMS patients or the control subjects, and higher abundance was presented in CL (74.6% in average) than in CU.

Among CV samples, we found the decrease of the *Lactobacillus* abundance and the appearance of *Veillonellaceae* (type V) (Fig. 1c). The case numbers of the type I, II, III, IV and V from EMS patients were 0, 23, 1, 11 and 1 respectively, while 2, 9, 0, 3 and 0 cases in control group. 3 type II CV samples of EMS patients shifted to type IV and V in CU, but no control cases changed. It demonstrated that a trend of *Lactobacillus* reducing was occurred in EMS patients. However, *Lactobacillus* was still the dominant in CV samples, accounting for 63.9% and 64.3% in the EMS and the control group, respectively. Although there was no significant difference in type II between EMS and control through the statistical analysis, type IV community in these two groups were significantly different (*p value* < 0.05).

In total, 26 ET samples from EMS patients and 11 from control were sequenced and moved to further analysis. The dominates fell into 4 types, including I, II, VI and VII (Fig. 1d). The ratio of type II in EMS cases was cut down more than 50% in ET when compared to CV. While in control, the ratio of *Lactobacillus* was at the same level all through the lower reproductive tract. Interestingly, type VI presented highest abundance among all types in ET samples, which was not detected in the lower reproductive tract samples. Besides, the proportion of type VI in EMS patients were significantly higher than that in the control group which further manifested the different microbial distribution occurred in EMS.

The composition of the microbiota in PF samples from two groups were presented in Fig. 1e. All samples in the PF have no distinct dominant bacteria and harbored a more complicate and diverse microbiota. We can barely find the existence of *Lactobacillus* in PF samples. Meanwhile, genus belongs to type IV represented a larger proportion in PF than in ET, which accounted for 34/36 (94.4%) and 11/14 (78.6%) in EMS and control group. Furthermore, genus *Comamonas* (type VII) appeared only in PF samples from both EMS and the control group among all the reproductive tract locations.

### Signature species in the reproductive tract of patients with EMS

**Fig. 2:**
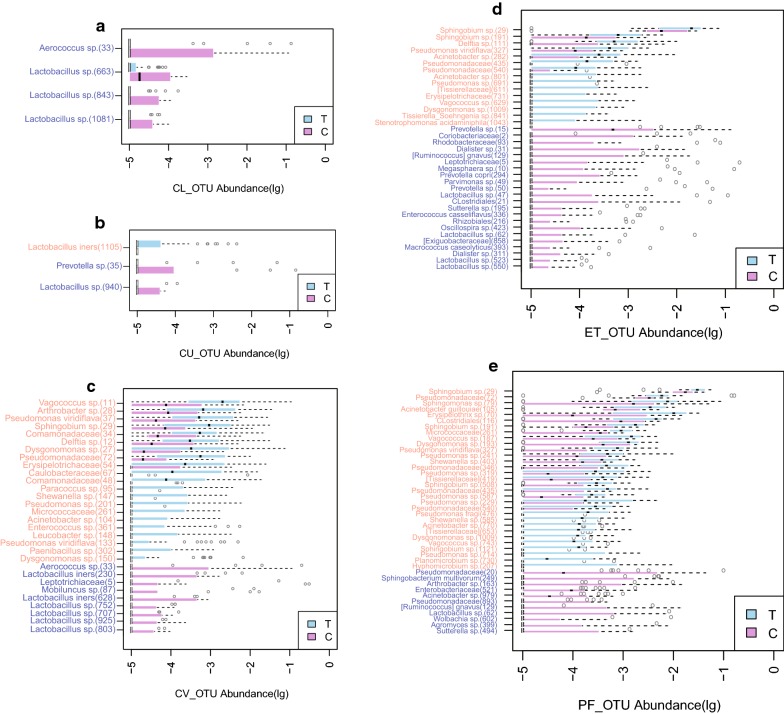
The signature OTUs identified in the different sites of female reproductive tract were shown, including **a** vagina (CL), **b** posterior vaginal fornix (CU), **c** cervical mucus (CV), **d** endometrium (ET) and **e** peritoneal fluid (PF). Blue bar and purple bar note higher abundancy were occurred in EMS patients and healthy women, representatively. The signature OTUs were defined by Wilcoxon-rank sum test with *p* value < 0.05

To dress a comprehensive understanding toward the microbial difference throughout the reproductive tract between EMS patients and non-EMS people, the signature OTUs were defined by Wilcoxon-rank sum test with p value < 0.05 (Fig. 2). In general, the OTUs with significant difference between two groups in the lower reproductive tract mostly belong to *Lactobacillus* species, which were considered as the probiotic in the vaginal. The various may result from the individual difference. In the CU samples, *Lactobacillus iners* was enriched in the EMS patients which indicated there may be some special pathway in this species associated with EMS. However, from CV, ET to PF, especially in PF, many species showed their specificity either in EMS samples or control ones.

As the microbiota composition of CL and CV was similar and mainly dominated by *Lactobacillus*, only 4 and 3 species with significant different abundance obtained from the analysis and most of them were *Lactobacillus* sp. (Fig. 2a, b). Among them, *Lactobacillus iners* which manifested higher abundance in control cases was worth raising here since it is one of the important species inhabited in the healthy female reproductive tract. While, for those did not belong to *Lactobacillus*, *Aerococcus* sp. (33) from CL and *Prevotella* sp. (35) from CU were enriched in EMS patients.

In CV, where located at the transition zone between lower and upper reproductive tract, *Vagococcus* sp. [11], *Arthrobacter* sp. (28), *Pseudomonas viridiflava* (37), *Sphingobium* sp. (29), *Comamonadaceae* (34) and *Delftia* sp. [12] at the species level were significantly enriched in the EMS group (p < 0.05), as well as family Pseudomonadaceae (72), Erysipelotrichaceae (54) and Caulobacteraceae (67). In the control group, *Lactobacillus* sp. remained its significant higher abundance (Fig. 2c). For the ET samples, signature species in EMS cases were *Sphingobium* sp. (29), *Sphingobium* sp. (191), *Delftia* sp. (111), *Pseudomonas viridiflava* (327) and *Acinetobacter* sp. (282). At family level, Pseudomonadaceae (435) was significantly enriched in the EMS group (p < 0.05) (Fig. 2d). Even so, more significant different OTUs enriched in PF samples from EMS group (p value < 0.05), including *Sphingobium* sp. (29), *Pseudomonadac*eae (72), *Sphingomonas* sp. (79), *Acinetobacter guillouiae* (105), *Erysipelothrix* sp. (70), *Clostridiales* (116), *Sphingobium* sp. (191), Micrococcaceae (261), *Vagococcus* sp. (187), *Dysgonomonas* sp. (193), P*seudomonas viridiflava* (327), *Shewanella* sp. (403), Pseudomonadaceae (346), *Pseudomonas* sp. (319), Tissierellaceae (419) and *Sphingobium* sp. (508) (Fig. 2e). Notably, *Sphingobium* sp. (29) and *Pseudomonas viridiflava* (327) were significantly enriched both in the ET and PF from EMS patients, which could identify as the microbial marker of EMS.

## Discussion

We here applied 16S rRNA gene sequencing to evaluate the microbial community along the female reproductive tract of the endometriosis patients, which confirmed the existence of the microorganism and distinct community composition in the lower third of vagina (CL), posterior vaginal fornix (CU), cervical mucus (CV), endometrium (ET) and peritoneal fluid (PF). After addressing the difference between EMS patient and non-EMS women, the microbiota structure was altered in EMS and signature species were determined.

The pathogenesis of EMS is complex, and there is still no perfect theory to explain its pathogenesis. Sampson proposed the transfusion theory of menstrual blood in the 19th century and this still been widely supported [19]. Liu and Lang believes the activity of endometrial cells determines the development of EMS [20]. However, a group of scientists followed the explanation of Khan, which is the bacterial contamination hypothesis due to the discovery of bacterial colonization in the endometrium of EMS patients [12, 15, 21]. In the current study, many significant different OTUs presented in the flora of ET and PF inferred the specific microbial community existed in EMS. According to the above three hypotheses and this study, the pathogenesis of EMS could start from the intrauterine chronic inflammation reaction induced by specific bacterial contamination, and the immune response accelerated the cell generation of endometrium, which led to the EMS. On the other hand, the retrograde menstrual blood could be the great substrate for bacteria in the pelvis, and the bacterial colonization could largely increase the risk of EMS. Hence, the understanding of the microbiota through all the female reproductive tract in the EMS patients is greatly contributes to the exploration of EMS pathogenesis.

Up to now, the research on the distribution of female reproductive flora is mainly focuses on vaginal microbiota or limited to small-scale exploratory on the single site [22–24]. Although the previous study has found the intrauterine bacterial colonization in EMS patients by 16S rRNA gene sequencing analysis, the study did not establish a control group [15]. In this study, a multi-site sample of the reproductive tract was collected from 36 EMS patients and 14 non-EMS patients by a strictly controlled sampling method under the surgery condition. The 16S rRNA gene sequencing analysis showed the microbiota difference but continuum along the female reproductive tract. The structure of the flora in the lower reproductive tract (CL, CU, CV) of the same individual is relatively stable, mainly dominated by *Lactobacillus* (type II). However, *Lactobacillus* was dramatically decreased in the upper reproductive tract of EMS patients and the flora type was shifted to a diverse one (type IV). In addition, the abundance of *Lactobacillus* was even lower in PF samples. The diversity of the community increased in upper reproductive tract by the occurrence and even dominance of *Pseudomonas*, *Acinetobacter* and *Vagococcus*. Therefore, the pattern of the distribution of genital tract flora in reproductive-age women is different either in the upper and lower reproductive tract or in the EMS and non-EMS subjects.

The signature species of EMS was selected to address the different microbiota in specific sites. Firstly, the proportion of *Lactobacillus* in the EMS patient’s lower reproductive tract (CL, CU) was less than control cases, and the case number of non-*Lactobacillus* dominated microbiota significantly increased in CV. In the control group, CL, CU and CV were consistently dominated by *Lactobacillus* sp., which was similar to the distribution of healthy women [25–27]. Meanwhile, signature OTUs gradually increased from lower to upper reproductive tract and reached the peak in pelvic cavity. These findings suggest a distinct genital tract microflora in EMS patient and provide a prediction for EMS-prone individuals. It is also worth noting that *Sphingobium* sp (29) and *Pseudomonas viridiflava* (327) were significantly enriched in ET and PF, revealing the abundance of these two species might play a key role in EMS pathogenesis. The endometrium and peritoneal fluid should also be regarded as represent sites to study the EMS reproductive tract microecology.

Although we depicted the relationship between microbiota or specific species and EMS, it still has its limitations. Firstly, a larger and multi-central cohort research is needed to further confirm the effect of the microbiota. Secondly, more healthy control cases should be included to exclude the interference of other gynecologic diseases. What’s more important, to investigate the functional network of these microbes and evaluate how the species interact with the host is the essential path to clinical applications.

## Conclusion

In summary, from lower to upper reproductive tract, a significant difference in the distribution of the microbiota began showing in the CV of EMS patients and gradually increased upward the reproductive tract. The microbiota in cervical samples is expected to be an indicator for the risk of catching EMS. The decreasing of *Lactobacillus* in vaginal flora and the increasing of signature OTUs in transaction zone (CV) and upper reproductive tract (ET, PF) of EMS patients reflect the EMS-associated alteration of microbial community, the participation of specific colonized bacteria in the EMS pathogenesis, as well as the relationship between microbiota and development of disease.

## Importance

We believe that our research provided valuable microbial data to the studies of female reproductive tract (FRT) and this is the first time of elaborating the detailed microbiota associated with EMS. The dynamic of microbiomes related with the micro-environment should be considered as a critical feature for future study of pathogenesis not only for EMS, but also for other FRT diseases.

## Data Availability

The datasets used and/or analyzed during the current study are available from the corresponding author on reasonable request.

## References

[1] Giudice LC (2010). Endometriosis. N Engl J Med.

[2] Haas D, Chvatal R, Reichert B, Renner S, Shebl O, Binder H, Wurm P, Oppelt P (2012). Endometriosis: a premenopausal disease? Age pattern in 42,079 patients with endometriosis. Arch Gynecol Obstet.

[3] Schneider A, Touloupidis S, Papatsoris AG, Triantafyllidis A, Kollias A, Schweppe KW (2006). Endometriosis of the urinary tract in women of reproductive age. Int J Urol.

[4] Medicine PCotASfR (2004). Endometriosis and infertility. Fertil Steril.

[5] Burney RO, Giudice LC (2012). Pathogenesis and pathophysiology of endometriosis. Fertil Steril.

[6] Attar E, Bulun SE (2005). Aromatase and other steroidogenic genes in endometriosis: translational aspects. Hum Reprod.

[7] Khan KN, Kitajima M, Hiraki K, Fujishita A, Sekine I, Ishimaru T, Masuzaki H (2008). Immunopathogenesis of pelvic endometriosis: role of hepatocyte growth factor, macrophages and ovarian steroids. Am J Reprod Immunol.

[8] Khan KN, Masuzaki H, Fujishita A, Kitajima M, Sekine I, Ishimaru T (2004). Differential macrophage infiltration in early and advanced endometriosis and adjacent peritoneum. Fertil Steril.

[9] Wira CR, Fahey JV, Sentman CL, Pioli PA, Shen L (2005). Innate and adaptive immunity in female genital tract: cellular responses and interactions. Immunol Rev.

[10] Khan KN, Kitajima M, Hiraki K, Fujishita A, Sekine I, Ishimaru T, Masuzaki H (2009). Toll-like receptors in innate immunity: role of bacterial endotoxin and toll-like receptor 4 in endometrium and endometriosis. Gynecol Obstet Invest.

[11] Fazeli A, Bruce C, Anumba D (2005). Characterization of toll-like receptors in the female reproductive tract in humans. Hum Reprod.

[12] Khan KN, Fujishita A, Hiraki K, Kitajima M, Nakashima M, Fushiki S, Kitawaki J (2018). Bacterial contamination hypothesis: a new concept in endometriosis. Reprod Med Biol.

[13] Khan KN, Kitajima M, Hiraki K, Yamaguchi N, Katamine S, Matsuyama T, Nakashima M, Fujishita A, Ishimaru T, Masuzaki H (2010). Escherichia coli contamination of menstrual blood and effect of bacterial endotoxin on endometriosis. Fertil Steril.

[14] Alexander JW, Boyce ST, Babcock GF, Gianotti L, Peck MD, Dunn DL, Pyles T, Childress CP, Ash SK (1990). The process of microbial translocation. Ann Surg.

[15] Khan KN, Fujishita A, Kitajima M, Hiraki K, Nakashima M, Masuzaki H (2014). Intra-uterine microbial colonization and occurrence of endometritis in women with endometriosis. Hum Reprod.

[16] Zengler K, Toledo G, Rappé M, Elkins J, Mathur EJ, Short JM, Keller M (2002). Cultivating the uncultured. Proc Natl Acad Sci.

[17] Chen C, Song X, Wei W, Zhong H, Dai J, Lan Z, Li F, Yu X, Feng Q, Wang Z (2017). The microbiota continuum along the female reproductive tract and its relation to uterine-related diseases. Nat Commun.

[18] Li F, Chen C, Wei W, Wang Z, Dai J, Hao L, Song L, Zhang X, Zeng L, Du H (2018). The metagenome of the female upper reproductive tract. GigaScience.

[19] Sampson JA (1927). Metastatic or embolic endometriosis, due to the menstrual dissemination of endometrial tissue into the venous circulation. Am J Pathol.

[20] Liu H, Lang JH (2011). Is abnormal eutopic endometrium the cause of endometriosis? The role of eutopic endometrium in pathogenesis of endometriosis. Med Sci Monit.

[21] Khan KN, Fujishita A, Masumoto H, Muto H, Kitajima M, Masuzaki H, Kitawaki J (2016). Molecular detection of intrauterine microbial colonization in women with endometriosis. Eur J Obstet Gynecol Reprod Biol.

[22] Kroon SJ, Ravel J, Huston WM (2018). Cervicovaginal microbiota, women’s health, and reproductive outcomes. Fertil Steril.

[23] Nelson DB, Rockwell LC, Prioleau MD, Goetzl L (2016). The role of the bacterial microbiota on reproductive and pregnancy health. Anaerobe.

[24] Ravel J, Gajer P, Abdo Z, Schneider GM, Koenig SS, McCulle SL, Karlebach S, Gorle R, Russell J, Tacket CO (2011). Vaginal microbiome of reproductive-age women. Proc Natl Acad Sci.

[25] Boris S, Barbés C (2000). Role played by lactobacilli in controlling the population of vaginal pathogens. Microbes Infect.

[26] Borges S, Silva J, Teixeira P (2014). The role of lactobacilli and probiotics in maintaining vaginal health. Arch Gynecol Obstet.

[27] Moreno I, Codoñer FM, Vilella F, Valbuena D, Martinez-Blanch JF, Jimenez-Almazán J, Alonso R, Alamá P, Remohí J, Pellicer A (2016). Evidence that the endometrial microbiota has an effect on implantation success or failure. Am J Obstet Gynecol.

